# Epidemiological and osteoarticular involvement sites’ characteristics of multiple osteoarticular tuberculosis: a scoping review

**DOI:** 10.1017/S095026882400150X

**Published:** 2025-01-21

**Authors:** Jian Zhou, Xuanjie Yang, Yong Hu, Shijun Li

**Affiliations:** 1 School of Public Health, the Key Laboratory of Environmental Pollution Monitoring and Disease Control, Ministry of Education, Guizhou Medical University, Guiyang, China; 2 Guizhou Center for Disease Control and Prevention, Guiyang, China

**Keywords:** age, clinical type, gender, involvement sites, multifocal osteoarticular tuberculosis

## Abstract

Multiple osteoarticular tuberculosis (MOT) represents an uncommon yet severe form of tuberculosis, characterized by a lack of systematic analysis and comprehension. Our objective was to delineate MOT’s epidemiological characteristics and establish a scientific foundation for prevention and treatment. We conducted searches across eight databases to identify relevant articles. Pearson’s chi-square test (Fisher’s exact test) and Bonferroni method were employed to assess osteoarticular involvement among patients of varying age and gender (α = 0.05). The study comprised 98 articles, encompassing 151 cases from 22 countries, with China and India collectively contributing 67.55% of cases. MOT predominantly affected individuals aged 0–30 years (58.94%). Pulmonary tuberculosis was evident in 16.55% of cases, with spinal involvement prevalent (57.62%). Significant differences were noted in trunk, spine, thoracic, and lumbar vertebrae involvement, as well as type I lesions across age groups, increasing with age. Moreover, significant differences were observed in upper limb bone involvement and type II lesions across age groups, decreasing with age. Gender differences were not significant. MOT primarily manifests in China and India, predominantly among younger individuals, indicating age-related variations in osteoarticular involvement. Enhanced clinical awareness is crucial for accurate MOT diagnosis, mitigating missed diagnoses and misdiagnoses.

## Introduction

Multiple osteoarticular tuberculosis (MOT) is a chronic infectious disease caused by *Mycobacterium tuberculosis* infection, characterized by extensive lesions in bone and joint tissues [1–3]. MOT typically affects multiple skeletal sites and joints, either simultaneously or consecutively, with common locations including the spine, pelvis, femoral head, shoulder joints, and knee joints [4]. Symptoms of MOT include joint pain, stiffness, redness, swelling, localized temperature elevation, restricted movement, and consequent bone deformation [5]. The diagnostic process for MOT commonly involves various imaging modalities such as X-ray, CT scan, and magnetic resonance imaging (MRI). Confirmation of the diagnosis may require bacterial testing and tissue biopsy [5–7].

The pathogenesis of bone and joint tuberculosis is believed to involve *M. tuberculosis* infection. Typically entering through the respiratory tract and disseminating via the circulatory system, the bacteria eventually colonize bone and joint tissues. Due to the relatively weak resistance of these tissues to bacterial invasion, inflammation, and destruction occur [4]. However, the pathogenesis of bone and joint tuberculosis in patients without pulmonary tuberculosis remains less understood. Furthermore, the clinical symptoms associated with this condition are atypical, making diagnosis challenging and prone to misdiagnosis and oversight [8–11].

Given its rarity, MOT cases are primarily reported as individual case reports in current literature. Establishing a diagnosis for this uncommon condition requires a comprehensive approach that includes systematic epidemiological and clinical evidence. Therefore, to address this gap in knowledge, we made efforts to collect information on MOT cases from the literature database, and the language types of literature for this scoping review were English and Chinese.

## Methods

The protocol for this systematic review was registered on the PROSPERO (International Prospective Register of Systematic Reviews) and assigned registration number CRD42023494495, which can be accessed at https://www.crd.york.ac.uk/PROSPERO/. As the study was based on previously published preliminary research, ethical approval and patient consent were not sought.

### Search strategy

Two reviewers (JZ and XJY) conducted searches in Embase, PubMed, Scopus, Web of Science, Cochrane Library, Chinese National Knowledge Infrastructure (CNKI), Wanfang, and VIP databases. The search deadline was set as 12 December 2023. A Boolean search strategy with subject words and free words was employed using keywords and MeSH or Emtree terms related to ‘multifocal’ and ‘bone and joint tuberculosis’ for each database (Supplementary Table 1: Search strategies).

### Eligibility criteria

Studies meeting the following criteria were included: (1) primarily consisting of case reports and original studies, (2) involving human subjects without discrimination based on age, gender, or geographical origin; and (3) requiring a diagnosis supported by comprehensive evidence from imaging, bacteriological, or biopsy sources. Exclusion criteria were (1) reviews, conference papers, abstracts, expert opinions, and repeated literature were excluded; (2) literature with unclear diagnosis, especially those without imaging and bacteriological evidence, was excluded; (3) infections due to nontuberculous *Mycobacteria* were excluded; and (4) literature without gender and age of patients was excluded.

### Quality assessment and data extraction

This study adhered to the rigorous guidelines of the Preferred Reporting Items for Systematic Reviews and Meta-analyses (PRISMA) [12]. In strict accordance with the diagnostic criteria for bone and joint tuberculosis, we considered imaging and bacteriological evidence as crucial tools for MOT diagnosis and assessed patient eligibility based on MOT determination conditions [4]. Two individuals (JZ and XJY) extracted the literature data, respectively, and finally, combined the data and verified the accuracy of the data. In cases where discrepancies arose between the two extractions, a thorough re-examination of the literature was conducted to obtain accurate data (Supplementary Table 2: Data collection evaluation form).

### Variable definitions

Using a standardized data abstraction form, we recorded the following information from articles that met our inclusion criteria: reporting time, country, gender, age, presence or absence of pulmonary tuberculosis, and sites of bone and joint involvement. MOT was defined as the simultaneous occurrence of two or more non-adjacent bone and/or joint lesions caused by *M. tuberculosis.* MOT was further categorized into three types: type I involved multiple spinal segments (or the same segment separated by at least one vertebral body), type II involved multiple joints only, and type III involved complex multiple bones including joints, spine, or other parts [4].

Age groups were divided into four categories: 0–20, 21–40, 41–60, and above 61 years old. The affected regions of the body were classified as skull bones, trunk bones (including ribs), upper limb bones (including shoulder girdle), lower limb bones (including pelvic girdle), and different segments of the spine such as thoracic vertebrae lumbar vertebrae cervical vertebrae sacral vertebrae. Proportions were used to describe and analyze specific indicators of bone involvement. Furthermore, the proportions of bone and joint involvement in different genders and age groups were calculated to examine potential differences.

### Statistical analysis

SPSS 26.0 software was used to analyze the extracted data. According to the conditions of the data, Pearson’s chi-square test or Fisher’s exact test was used to analyze the differences in the proportion of bone and joint involvement in different genders and ages. If the difference in the proportion between groups was statistically significant, Bonferroni method was used for pairwise comparison between groups. In addition, the chi-square test for trend was used to analyze the trend of proportion in different age groups. The test level α = 0.05.

### Patient and public involvement

The Ethics Committee of the Guizhou Center for Disease Control and Prevention considered that the ethical approval and consent to participant were not relevant to this study, ethical approval is not required for this review, as it will exclusively use data from published studies.

## Results

### Description of studies

**Figure 1.**
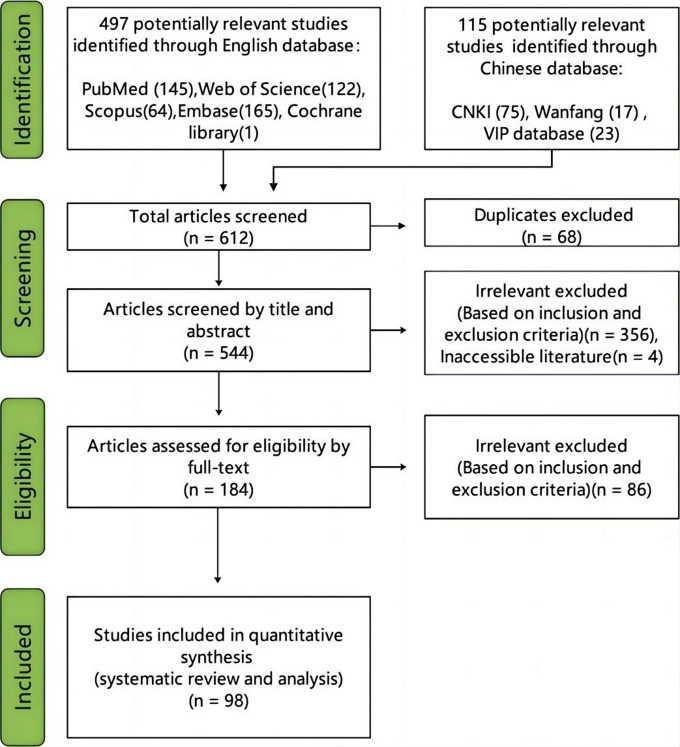
PRISMA flow diagram for study selection.

After conducting an extensive literature search, a total of 612 articles were initially retrieved from various databases including PubMed (145), Web of Science (122), Scopus (64), Embase (165), Cochrane library (1), CNKI (75), Wanfang (17), and VIP database (23). Among them, 68 articles were excluded due to duplication, while 356 articles did not align with the study’s objectives based on their title and abstract content. Additionally, 4 articles were inaccessible for various reasons (Figure 1). Subsequently, the remaining 184 articles underwent full-text evaluation resulting in the exclusion of another 86 articles. Ultimately, 98 articles were finally included in our study [1, 13–109], a total of 151 cases (Supplementary Table 2) .

### Basic characteristics of MOT cases

**Figure 2.**
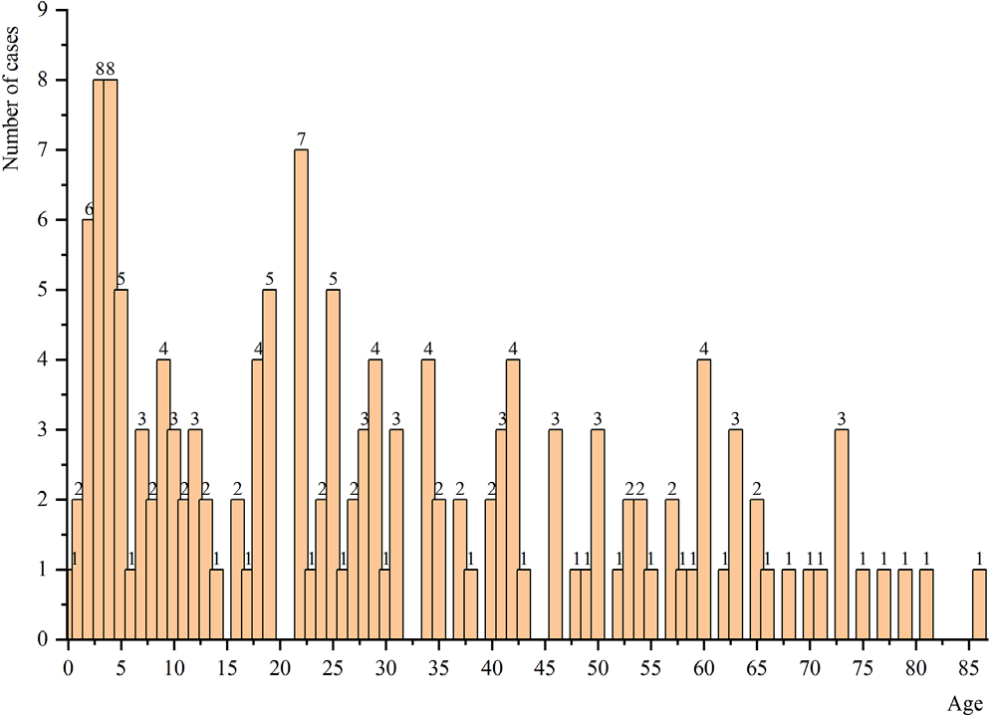
Age distribution of MOT cases.

These 151 cases of MOT were reported between 1958 and 2023, with ages ranging from 7 months to 86 years (median: 25; mean: 30), the highest proportion of cases was observed in the 0–5 years, accounting for 19.87%, with a gradual decline in case numbers noted as age increased (Figure 2). Of the total, there were 89 males and 62 females (male/female = 1.44), distributed across 22 countries worldwide, with China accounting for the largest proportion at 35.10% followed by India at 32.45%. Patients aged between 0 and 10 years accounted for the highest proportion(28.48%), while those aged between 0 and 30 years accounted for nearly 60%. Pulmonary tuberculosis was present in 25 patients with MOT (male/female = 2.57), representing 16.55% of all cases. In terms of bone involvement sites, trunk bones were most commonly affected (63.58%), with spine involvement being predominant (90.63%) among them. Lower limb bone involvement was also common (60.93%). Spinal involvement occurred in over half of all cases (57.62%), with thoracic vertebrae being the most frequently involved site (42.38%) followed by lumbar vertebrae (36.42%). In terms of clinical classification, type I represented 13.91%, while types II and III accounted for 41.72% and 44.37%, respectively (Table 1).

**Table 1.** Basic characteristics of patients with MOT (N = 151)

Category	Number of case	Proportion (%)	Category	Number of case	Proportion (%)
Gender	151	100.00	Period of cases	151	100.00
Male	89	58.94	1958 ~ 1999	28	18.54
Female	62	41.06	2000 ~ 2009	33	21.85
Age	151	100.00	2010 ~ 2019	64	42.38
0 ~ 10	43	28.48	2020 ~ 2023	26	17.22
11 ~ 20	20	13.25	Country	151	100.00
21 ~ 30	26	17.22	China	53	35.10
31 ~ 40	14	9.27	India	49	32.45
41 ~ 50	16	10.60	Turkey	11	7.28
51 ~ 60	14	9.27	Iran	4	2.65
61 ~ 70	9	5.96	Pakistan	4	2.65
71 ~ 80	7	4.64	South Africa	4	2.65
80 ~ 90	2	1.32	USA	4	2.65
Pulmonary tuberculosis	151	100.00	Morocco	3	1.99
Yes	25	16.55	UK	3	1.99
No	83	54.97	Filipino	2	1.32
Unknown	43	28.48	Poland	2	1.32
Site of involvement	151	100.00	Tunisia	2	1.32
Skull	22	14.57	Afghanistan	1	0.66
Trunk	96	63.58	Colombia	1	0.66
Upper limb	69	45.70	Indonesia	1	0.66
Lower limb	92	60.93	Israel	1	0.66
Site of spinal involvement	87	57.62	Japan	1	0.66
Thoracic vertebrae	64	42.38	Korea	1	0.66
Lumbar vertebrae	55	36.42	Malaysia	1	0.66
Cervical vertebrae	23	15.23	Portugal	1	0.66
Sacral vertebrae	15	9.93	Senegalese	1	0.66
Type of MOT	151	100.00	Spain	1	0.66
Type I	21	13.91			
Type II	63	41.72			
Type III	67	44.37			

### Distribution characteristics of osteoarticular involvement in different ages

**Figure 3.**
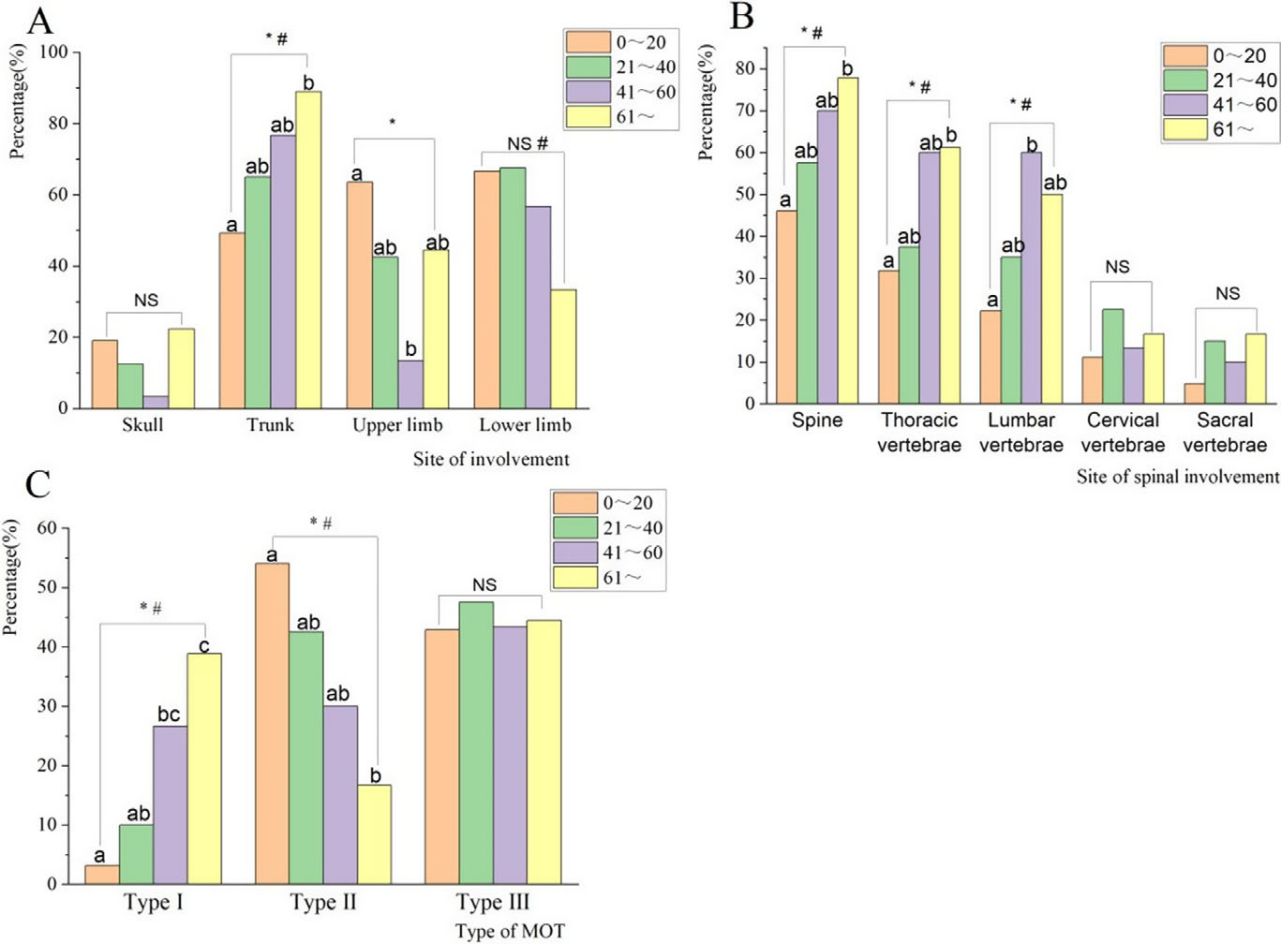
(a) Distribution characteristics of osteoarticular involvement sites in different ages. (b) Distribution characteristics of spinal involvement sites in different age groups. (c) Characteristics of clinical types of MOT in different age groups. The letters represent pairwise comparisons between groups, and the different letters between groups represent statistically significant differences (*P* < 0.05); ‘NS’ indicates no statistically significant difference between groups (*P* > 0.05); ‘*’ indicates statistically significant differences between groups (*P* < 0.05); ‘#’ represents trend chi-square test with statistical significance (*P* < 0.05).

There was no statistically significant difference in the proportion of skull involvement among different age groups (*P* = 0.13). However, both MOT cases over 61 years of age and those aged 0–20 years had a higher proportion of skull involvement. A significant difference was observed in the proportion of trunk osteoarticular involvement between different age groups (*P* = 0.005), with the most pronounced difference found between the 0- to 20-years-old group (49.21%) and the over 60-years-old group (88.89%) (*P* < 0.05). Furthermore, there was an increasing trend in the proportion of trunk osteoarticular involvement with advancing age (*P* < 0.001). Significant differences were identified in the proportion of upper limb osteoarticular involvement among different age groups (*P* < 0.001), particularly within the 0- to 20-years-old group (63.49%) and the 41- to 60–years-old group (13.33%). No significant difference was observed in the proportion of lower limb osteoarticular involvement across different age groups (*P* = 0.055), but there was a decreasing trend noted as age increased (*P* = 0.018) (Figure 3a).

### Distribution characteristics of spinal involvement in different ages

There was a significant difference in the proportion of spinal involvement among different age groups (*P* = 0.039), particularly between the 0–20 age group (46.03%) and those over 61 years old (77.78%) (*P* < 0.05). The proportion of spinal involvement increased with age (*P* = 0.004). Significant differences were observed in the proportion of thoracic vertebrae involvement among different age groups (*P* = 0.021), especially between the 0–20 age group (31.75%) and those over 61 years old (61.11%) (*P* < 0.05). The proportion of thoracic vertebrae involvement also increased with age (*P* = 0.003). Furthermore, there was a significant difference in the proportion of lumbar vertebrae involvement among different age groups (*P* = 0.003), particularly between the 0–20 years old and those aged from 41 to 60 years old(60% vs. 22%, respectively; *P* < 0.05), and the proportion of lumbar vertebrae involvement increased with age (*P* = 0.001), while no significant difference was found for cervical or sacral vertebrae involvement across all ages (*P* > 0.05) (Figure 3b).

### Clinical classification characteristics in different ages

There was a significant difference in the distribution of type I cases across different age groups (*P* < 0.001), particularly within the 0–20 (3.17%), 21–40 (10.00%), and over 61 (38.89%) age groups (*P* < 0.05). Moreover, there was an upward trend in the proportion of type I cases with advancing age (*P* < 0.001). The proportion of type II cases exhibited a notable variation among various age groups as well (*P* = 0.017), especially between individuals aged 0- to 20-years old (53.97%) and those above 61 years old (16.67%) (*P* < 0.05). Furthermore, there was an inverse relationship between the proportion of type II cases and increasing age (*P* = 0.001). Conversely, no statistically significant difference existed in the distribution of type III cases across different age groups (*P* = 0.973) (Figure 3c).

### Distribution characteristics of osteoarticular involvement in different genders

**Figure 4.**
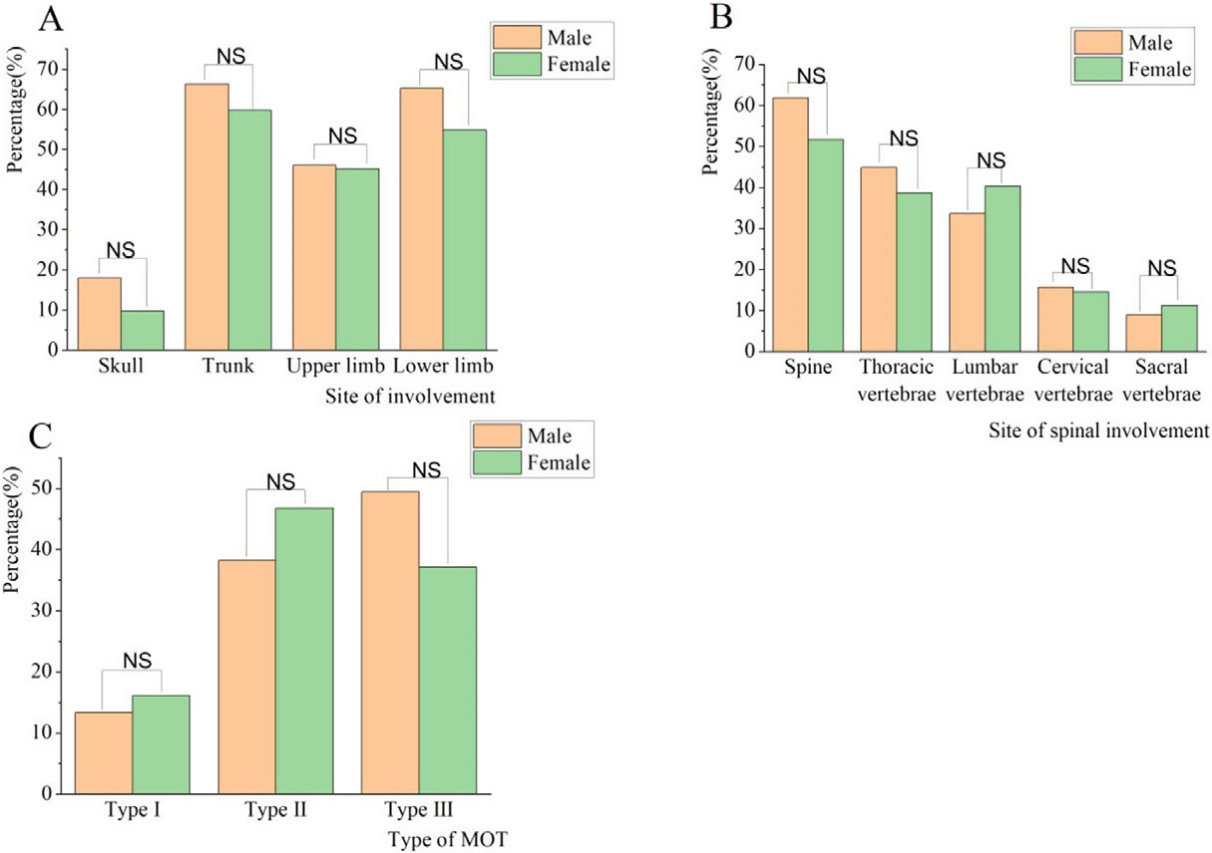
(a) Distribution characteristics of osteoarticular involvement sites in different genders. (b) Distribution characteristics of spinal involvement sites in different genders. (c) Clinical classification in different genders. ‘NS’ indicates no statistically significant difference between groups (*P* > 0.05).

Although there was no significant difference in the proportion of skull, trunk, upper limb, and lower limb osteoarticular involvement between male and female patients with MOT (*P* > 0.05), it was noted that the proportion of osteoarticular involvement was greater in males than in females, especially in the skull and lower limb bones and joints (Figure 4a).

### Distribution characteristics of spinal involvement in different genders

Even though there was no statistically significant difference in the proportion of spinal, thoracic, lumbar, cervical, and sacral vertebrae involved in MOT cases by gender (*P* > 0.05), it is noteworthy that male(61.80%) exhibited an overall higher proportion of spinal involvement compared to female(51.61%). However, females demonstrated a greater involvement of lumbar and sacral vertebrae than males (Figure 4b).

### Clinical classification characteristics in different genders

The proportions of type I, type II, and type III cases did not exhibit any significant differences across different genders (*P* > 0.05) (Figure 4c).

## Discussion

Among tuberculosis cases reported by the World Health Organization (WHO) in 2020, 18% were extrapulmonary, with incidence rates ranging from 9% in the Western Pacific Region to 24% in the Eastern Mediterranean Region [110]. Numerous studies have demonstrated an increasing trend in the proportion of extrapulmonary tuberculosis [111, 112]. Bone tuberculosis represents a prevalent form of extrapulmonary tuberculosis, accounting for approximately 10–15% of all cases [113]. A study has reported that in Guangxi province in China, bone and joint tuberculosis accounted for as high as 27.20% of extrapulmonary cases [114].

Conversely, MOT is a rare yet clinically severe manifestation outside the lungs that manifests through pain, tenderness, and activity limitations along with specific and general symptoms depending on disease location, stage, and severity [115, 116]. The physical pain endured by patients with multiple bone tuberculosis coupled with mental and financial stress is unimaginable. This study aimed to elucidate the epidemiological characteristics of MOT and distribution patterns of bone involvement.

We found that the reported MOT cases have increased year by year, suggesting that there may be more MOT cases that have not been detected or reported, which further indicates the importance of the diagnosis and treatment of MOT. Our findings reveal that MOT cases are predominantly concentrated in developing countries such as China and India which also bear the highest burden of TB globally [117, 118]. Furthermore, there is a higher prevalence among males compared to females aligning with gender-specific characteristics observed for tuberculosis infections [119, 120]. Concurrently, the reporting rate for MOT cases has been consistently increasing over time indicating a need for greater attention and research focus.

Our study also presents some notable findings. First, the age distribution of MOT cases primarily concentrates between 0 and 30 years old, particularly within the 0–10 year age group, indicating a tendency for MOT occurrence in younger individuals. Second, children aged 0–5 years represented the highest number of cases; however, it remains unclear whether this is related to their immune systems or BCG vaccination, as evidence in this area is lacking. This underscores the need for increased attention and research on the risk of MOT in children. The proportion of spinal involvement in MOT cases reaches 57.62%, with thoracic vertebrae being predominantly affected, aligning with previous studies [121, 122].

Age stratification analysis reveals that both younger and older age groups exhibit a higher likelihood of skull involvement, while upper limb bone involvement is more prevalent among younger individuals. Notably, lower limb bone involvement decreases with age, while trunk bones become increasingly affected, especially the thoracic and lumbar vertebrae in the spine. Furthermore, gender differences analysis indicates that males are more prone to skull and lower limb bone involvement, whereas females demonstrate a higher incidence of lumbar and sacral spine involvement; however, further clinical evidence is required to substantiate these observations.

Our study highlights that MOT predominates in developing countries such as China and India. The observed trend towards younger ages at MOT onset along with distinct patterns of bone involvement based on age and gender underscore the importance of considering these characteristics during future clinical diagnosis processes to minimize missed or incorrect diagnoses of MOT. These significant findings warrant additional investigation to elucidate underlying reasons behind these observations aiming at reducing the prevalence of MOT and enhancing cure rates for multiple bone tuberculosis.

### Limitations

The primary limitation of our study lies in the insufficient availability of clinical information, such as comorbidities and immune function, for a significant number of MOT cases, thereby impeding further statistical analysis. Furthermore, certain literature sources were inaccessible due to various reasons. Finally, only Chinese and English literature could be accessed while literature in other languages remained unattainable.

## Conclusion

MOT cases are predominantly reported in developing countries, with a higher prevalence among individuals aged 0–30 years. Spinal involvement is the most prevalent (57.62%), while there is a decrease in lower limb bone involvement and an increase in trunk bone involvement with age, particularly affecting the thoracic and lumbar vertebrae. Males exhibited a greater likelihood of skull and lower limb bone involvement, whereas females were more prone to lumbar and sacral vertebrae involvement.

## Supporting information

Zhou et al. supplementary material 1 Zhou et al. supplementary material

Zhou et al. supplementary material 2 Zhou et al. supplementary material

## Data Availability

The original contributions presented in the study are included in the article/Supplementary Information. Further inquiries can be directed to the corresponding author.

## Supplementary material

The supplementary material for this article can be found at http://doi.org/10.1017/S095026882400150X.
