# Supplementary material for: Epidemiological and osteoarticular involvement sites’ characteristics of multiple osteoarticular tuberculosis: a scoping review
Source: Epidemiol Infect. 2025 Jan 21;153:e26. doi: 10.1017/S095026882400150X (PMC11869084; doi:10.1017/S095026882400150X)
Supplement: Zhou et al. supplementary material 1 — Zhou et al. supplementary material [file S095026882400150Xsup001.docx]

**Supplementary Table 1. Search strategy**

**Subject term** 1: Multifocal

**Free word 1:**Choroiditis, Multifocal

**Subject term** 2: Bone and joint tuberculosis

**Free word 2**: Osteoarticular Tuberculoses; Osteoarticular Tuberculoses; Tuberculoses, Osteoarticular;Bone Tuberculosis;Bone Tuberculosis;Bone Tuberculoses;Tuberculoses, Bone; Tuberculosis, Bone;Joint Tuberculosis ;Joint Tuberculoses; Tuberculoses, Joint; Tuberculosis, Joint.

**Search strategy：**

((Multifocal[Title/Abstract]) OR (Choroiditis, Multifocal[Title/Abstract])) AND ((((((((((((Bone[Title/Abstract] AND joint tuberculosis[Title/Abstract]) OR (Osteoarticular Tuberculoses[Title/Abstract])) OR (Osteoarticular Tuberculosis[Title/Abstract])) OR (Tuberculoses, Osteoarticular[Title/Abstract])) OR (Bone Tuberculosis[Title/Abstract])) OR (Bone Tuberculoses[Title/Abstract])) OR (Tuberculoses, Bone[Title/Abstract])) OR (Tuberculosis, Bone[Title/Abstract])) OR (Joint Tuberculosis[Title/Abstract])) OR (Joint Tuberculoses[Title/Abstract])) OR (Tuberculoses, Joint[Title/Abstract])) OR (Tuberculosis, Joint[Title/Abstract]))
