# Supplementary material for: Epidemiological and osteoarticular involvement sites’ characteristics of multiple osteoarticular tuberculosis: a scoping review
Source: Epidemiol Infect. 2025 Jan 21;153:e26. doi: 10.1017/S095026882400150X (PMC11869084; doi:10.1017/S095026882400150X)
Supplement: Zhou et al. supplementary material 2 — Zhou et al. supplementary material [file S095026882400150Xsup002.pdf]

Database	Study pirod	Publication time	Country	Author/time	tittle	Gender	Age	Pulmonary tuberculosis (past or present)	Skull	Trunk bones	Limb bones	Upper limb bones	Lower limb bones	Spine	Thoracic vertebrae	Lumbar vertebrae	Cervical vertebrae	Sacral vertebrae	rib	Classification of multiple bone tuberculosis	Sites of bone and joint involvement	Sites of bone and joint involvement	Sites of bone and joint involvement	Sites of bone and joint involvement	Sites of bone and joint involvement	Sites of bone and joint involvement	Sites of bone and joint involvement	Sites of bone and joint involvement	Sites of bone and joint involvement	Sites of bone and joint involvement	Sites of bone and joint involvement	Sites of bone and joint involvement
Pubmed		2011	India	Nazima Haider	Tubercular dactylitis and multifocal osteoarticular tuberculosis- two rare cases of extrapulmonary tuberculosis	man	4	yes	deny	deny	yes	yes	yes	deny	deny	deny	deny	deny	deny	Type II	Right side Four finger bones	offside humerus	offside ulna	right crus of diaphragm sphenoid	Right side One metatarsal							
Embase scopus wos		2017	India	Pravat Thatoi	Multifocal Tubercular Dactylitis: A Rare Presentation of Skeletal T uberculosis in an Adult	man	30	deny	deny	deny	yes	yes	yes	deny	deny	deny	deny	deny	deny	Type II	right crus of diaphragm digitus secundus pedis	the left hand big toe	the left hand index finger And the little finger	the right hand index finger And the little finger								
Pubmed		1999	Turkey	A.Arslan	Multifocal bone tuberculosis presenting as a breast mass: CT and MRI findings	woman	18	deny	deny	yes	deny	deny	deny	yes	yes	deny	deny	deny	yes	Type III	sternum	thoracic vertebra	rib									
Embase scopus wos		2011	India	A.R.Nataraj	Multifocal skeletal t uberculosis presenting as periarthritis of shoulder joint: a case report	woman	29	deny	deny	yes	yes	yes	yes	yes	yes	yes	deny	yes	yes	Type III	Right shoulder joint	offside humerus	rear sixth rib	sacrum	bilateral ilium	bilateral pubis	fl	lumbar vertebra	thoracic vertebra			
Embase scopus wos		2012	Afghanistan	Afzali H	A case report of disseminated multi focal bone tuberculosis	woman	16	yes	deny	yes	deny	yes	yes	yes	yes	yes	deny	deny	deny	Type III	lumbar vertebra	thoracic vertebra	right crus of diaphragm phalanx									
Embase scopus wos		1994	USA	ALAIN EID	M ultifocal Musculoskeletal Cystic Tuberculosis Without Systemic Man ifestat i ons	man	34	deny	deny	yes	yes	deny	yes	deny	deny	deny	deny	deny	deny	Type II	offside ilium	Left clavicle										
Pubmed		2023	Poland	Aleksandra Opala	A Successful Bisphosphonates Monot herapy in Spinal Form of Paediatric Chronic Recurrent Multifocal Osteomyelitis (CRMO)—Case Report	woman	9	deny	deny	yes	deny	deny	deny	yes	yes	yes	deny	deny	deny	Type I	lumbar vertebra	thoracic vertebra										
Pubmed		2014	Turkey	Ali Gur	Multifocal skeletal tuberculosis presenting asparaplegia: A case report	man	24	deny	deny	yes	yes	yes	yes	yes	yes	yes	deny	yes	yes	Type III	lumbar vertebra	thoracic vertebra	sternum	left side The ribs	left side ilium	Left shoulder shoulder blade	Left 1st Five ribs	sacral	left side Sacroiliac region			
Pubmed	2005	2009	India	AN Aggarwal	Tubercular osteomyelitis ofthe clavicle: a report of four cases	woman	22	yes	deny	yes	yes	yes	yes	deny	deny	deny	deny	deny	deny	Type II	Right clav	the left hand	Double sac ilium	the left hand								

																					icle	carpal	joint	articulatio carpi								
Pubmed		2011	India	Anil Agarwal	Multifocal osteoarticular tuberculosis in children	woman	1	unknown	deny	deny	yes	deny	yes	deny	deny	deny	deny	deny	deny	Type Ⅱ	Left elbow	carpal	second metacarpal bone									
Pubmed		2011	India	Anil Agarwal	Multifocal osteoarticular tuberculosis in children	woman	3	unknown	deny	deny	yes	yes	yes	deny	deny	deny	deny	deny	deny	Type Ⅱ	Right elbow				Right 1 One metatarsal							
Pubmed		2011	India	Anil Agarwal	Multifocal osteoarticular tuberculosis in children	man	3	unknown	deny	deny	yes	yes	yes	deny	deny	deny	deny	deny	deny	Type Ⅱ	Left food Point near The terminal phalanx				Left fifth metatarsal							
Pubmed		2011	India	Anil Agarwal	Multifocal osteoarticular tuberculosis in children	woman	3	unknown	deny	deny	deny	deny	deny	deny	deny	deny	deny	deny	deny	Type Ⅱ					Right tibia	Right side One metatarsal						

Pubmed		2011	India	Anil Agarwal	Multifocal osteoarticular tuberculosis in children	woman	4	unknown	deny	deny	yes	yes	yes	deny	deny	deny	deny	deny	deny	Type II	Right food Point near The terminal phalanx				Right side Five legs phalanx near-end							
Pubmed		2011	India	Anil Agarwal	Multifocal osteoarticular tuberculosis in children	woman	4	unknown	deny	deny	yes	yes	yes	deny	deny	deny	deny	deny	deny	Type II	Left ulna	Left 1st One metacarpal bone	Right side Second metacarpal		Left tibia	Right calcaneus						
Pubmed		2011	India	Anil Agarwal	Multifocal osteoarticular tuberculosis in children	man	4	unknown	deny	deny	yes	yes	yes	deny	deny	deny	deny	deny	deny	Type II	Right 1 Five metacarpals				Right femur							
Pubmed		2011	India	Anil Agarwal	Multifocal osteoarticular tuberculosis in children	man	4	unknown	deny	deny	yes	deny	yes	deny	deny	deny	deny	deny	deny	Type II	the right hand third metacarpal bone	the left hand index finger near-end phalanx	the left hand nameless Point near The terminal phalanx									
Pubmed		2011	India	Anil Agarwal	Multifocal osteoarticular tuberculosis in children	man	5	unknown	deny	yes	yes	yes	yes	yes	deny	deny	yes	deny	deny	Type III	Right humerus	Right radius Bone and osteoepiphysis	Left ulna	Left 1st three harmonies fifth metacarpal bone	Left					cervical vertebra		
Embase scopus wos		2014	India	Anil Agarwal	LYTIC LESIONS OF DISTAL RADIUS IN CHILDREN: A RARE TUBERCULAR PRESENTATION	man	5	deny	deny	yes	yes	yes	yes	yes	deny	deny	yes	deny	deny	Type III	Left 1st Three metacarpals	Left 1st Five metacarpals	Left tibia	Left humerus	Left ulna	cervical vertebra						
Pubmed		2011	India	Anil Agarwal	Multifocal osteoarticular tuberculosis in children	man	7	unknown	deny	deny	yes	deny	yes	deny	deny	deny	deny	deny	deny	Type II	Right elbow	Right wrist bone										
Pubmed		2011	India	Anil Agarwal	Multifocal osteoarticular tuberculosis in children	man	8	unknown	deny	deny	yes	yes	yes	deny	deny	deny	deny	deny	deny	Type II	Left 1st Five metacarpals				Right 1st One metatarsal							
Pubmed		2011	India	Anil Agarwal	Multifocal osteoarticular tuberculosis in children	man	9	unknown	deny	yes	deny	yes	yes	yes	yes	deny	deny	deny	deny	Type III					fl					thoracic verte		

																														bra		
Pubmed		2011	India	Anil Agarwal	Multifocal osteoarticular tuberculosis in children	man	10	unknown	deny	yes	yes	yes	yes	yes	deny	yes	deny	deny	deny	Type III	Left humerus	Two ulna bones	the right hand index finger phalanx	the right hand middle finger phalanx	Right femur	two flanks shin bone	two flanks huckle-bone	two flanks calcaneus	two flanks first metatarsal bones	lumbar vertebra		
Embase scopus wos		2014	India	Anil Agarwal	LYTIC LESIONS OF DISTAL RADIUS IN CHILDREN: A RARE TUBERCULAR PRESENTATION	man	10	yes	deny	yes	yes	yes	yes	yes	deny	yes	deny	deny	deny	Type III	Left humerus	two flanks ulna	the right hand phalanx	Right femur	bilateral shin bone	two flanks huckle-bone	two flanks Root heel bone	lumbar vertebra	bilateral first metatarsal bones			
Pubmed		2011	India	Anil Agarwal	Multifocal osteoarticular tuberculosis in children	woman	11	unknown	deny	yes	deny	yes	yes	yes	yes	deny	deny	deny	deny	Type III					Right tibia	Left big Weak legs organization abscessus	thoracic vertebra					
Pubmed		2011	India	Anil Agarwal	Multifocal osteoarticular tuberculosis in children	woman	12	unknown	deny	yes	yes	deny	yes	yes	yes	deny	deny	deny	deny	Type III	Left elbow	thoracic vertebra										
Pubmed		2011	India	Anil Agarwal	Multifocal osteoarticular tuberculosis in children	man	14	unknown	deny	deny	yes	yes	yes	deny	deny	deny	deny	deny	deny	Type II	Right radius				Left calcaneus							
Embase scopus wos		2017	India	Anirban Manda	The many faces of tub erculosis	man	3	deny	yes	deny	deny	yes	yes	deny	deny	deny	deny	deny	deny	Type II	Right tibia	os prefrontale	offside cheek bone									
Pubmed		1996	India	Anju Aggarwal	Multifocal Cystic Bone Tuberculosis with Lupus Vulgaris and Lymphadenitis	man	2	yes	deny	yes	yes	yes	yes	yes	yes	deny	deny	deny	deny	Type III	thoracic vertebra	both hands metacarpal bone	tibiofibula	metatarsal bones								

Pubmed		2006	India	Anuj Jaiswal	Multifocal skeletal TB or disseminated boneTB	woman	38	deny	deny	yes	deny	yes	yes	yes	yes	yes	deny	deny	deny	Type III	offside pubis	lumbar vertebra	thoracic vertebra								
Pubmed		2006	India	Anuj Jaiswal	Multifocal skeletal TB or disseminated boneTB	man	50	deny	deny	yes	deny	yes	yes	yes	yes	deny	deny	deny	deny	Type III	left side pubis	thoracic vertebra									
Pubmed		2005	India	Ashok Marudanayagam	MULTIFOCALSKELETAL TUBERCULOSIS: A REPORT OF THREE CASES	man	19	deny	deny	yes	deny	yes	yes	yes	yes	yes	deny	deny	deny	Type III	thoracic vertebra	lumbar vertebra	offside Sacroiliac joint	sternum							
Pubmed		2005	India	Ashok Marudanayagam	MULTIFOCALSKELETAL TUBERCULOSIS: A REPORT OF THREE CASES	man	41	deny	deny	yes	deny	yes	yes	yes	yes	deny	deny	deny	yes	Type III	thoracic vertebra	Right side 4 5 ribs	lumbar vertebra	left ilium	Right 1st One toe bone	left side Sacroiliac joint	offside first Metatarsophalangeal joint				
Pubmed		2005	India	Ashok Marudanayagam	MULTIFOCALSKELETAL TUBERCULOSIS: A REPORT OF THREE CASES	woman	55	unknown	deny	yes	deny	deny	deny	yes	yes	deny	deny	deny	deny	Type III	thoracic vertebra	sternum	Left sacrum Iliac joint								
Pubmed		2001	India	B.S.Morris	Multifocal musculoskeletal tuberculosisin children: appearances on computed tomography	man	2	deny	yes	deny	yes	deny	yes	deny	deny	deny	deny	deny	deny	Type II	Left shoulder shoulder blade	occipital bone									
Pubmed		2001	India	B.S.Morris	Multifocal musculoskeletal tuberculosisin children: appearances on computed tomography	man	6	deny	yes	yes	yes	deny	yes	yes	yes	deny	deny	deny	deny	Type III	thoracic vertebra	Left shoulder shoulder blade	the parietal bone								
Pubmed		2001	India	B.S.Morris	Multifocal musculoskeletal tuberculosisin children: appearances on computed tomography	woman	9	yes	deny	yes	deny	deny	deny	deny	deny	deny	deny	deny	deny	Type II	os prefrontale	sternum	Rib soft articulatio								
Pubmed		2001	India	B.S.Morris	Multifocal musculoskeletal tuberculosisin children: appearances on computed tomography	woman	13	deny	deny	yes	deny	deny	deny	yes	yes	deny	deny	deny	yes	Type III	offside fifth Root ribs	thoracic vertebra									
Pubmed		2018	India	Banavasi Shanmukha Girisha	A Case of Multifocal Tuberculosis InvolvingSkin and Bone: An Uncommon Presentation	woman	22	deny	deny	deny	yes	yes	yes	deny	deny	deny	deny	deny	deny	Type II	left crus phalanx	the right hand Thumb Phalanges	artifice joint								
Pubmed		2008	Turkey	Betul Sezgin	CONCOMITANT CUTANEOUS METASTATIC TUBERCULOUS ABSCESSES AND MULTIFOCAL SKELETAL TUBERCULOSIS	woman	5	deny	deny	deny	yes	yes	yes	deny	deny	deny	deny	deny	deny	Type II	left side fourth metac	offside ulna near-end	offside humerus near-	left side perone far-	bilateral calcaneus	left side shin bone far-					

																					arpal bone		end	end		end						
Pubmed		2005	USA	BINDESH	Multifocal Osteoarticular Tuberculosis	man	25	unknown	deny	deny	yes	yes	yes	deny	deny	deny	deny	deny	deny	Type II	two flanks knee–joint	Right side Two and a half fifth metacarpal bone	the left hand second And so on tridactylism	Left radius	offside shin bone	offside perone						
Pubmed		2022	Turkey	BURAK GÜNAY	A Rare Presentation of Tuberculosis: Multifocal Skeletal and Multisystemic Disease Evaluated with Imaging Findings	woman	27	deny	deny	yes	deny	deny	deny	yes	deny	yes	deny	deny	deny	Type I	lumbar vertebra											
Pubmed		2020	China	Congxiao Wang	Multifocal tuberculosis simu lating a cancer_ x005f a case report	man	18	deny	deny	yes	deny	yes	yes	yes	yes	deny	deny	yes	deny	Type III	thoracic vertebra	Right side Eight ribs	sacral	left side ilium								
Pubmed	1991	1998	Filipino	David S.Chang	Primary multifocal tuberculous osteomyelitis with involvement of the ribs	man	31	deny	deny	yes	deny	deny	deny	yes	deny	deny	yes	deny	yes	Type III	Left 1st Nine ribs	offside radix arcus vertebrae	cervical vertebra									

Pubmed	1995	1998	Senegalese	David S.Chang	Primary multifocal tuberculous osteomyelitis with involvement of the ribs	man	34	deny	deny	yes	yes	yes	yes	yes	yes	deny	deny	deny	yes	Type III	Left shoulder shoulder blade	left side Posteriorly Five ribs	left side ilium	left side Posterior Seven ribs	left side Back inside Side view Eight ribs	offside Former Foreign Minister Side1 Five ribs	offside acetabular bone	thoracic vertebra				
Pubmed		2008	India	Dhananjaya Sabat	SCAPHOLUNATE DISSOCIATION: A RARE PRESENTATION OF TB WRIST IN A CASE OF MULTI-FOCAL SKELETAL TUBERCULOSIS	woman	13	deny	deny	deny	yes	yes	yes	deny	deny	deny	deny	deny	deny	Type II	the left hand scapholunatum	the left hand articulatio carpi	radius	ulna	Right clavicle	right crus of diaphragm first metacarpal bone						
Pubmed		2006	Turkey	Duygu Ozol	Active Pulmonary Tuberculosis with Vertebraand Rib Involvement: Case Report	man	26	yes	deny	yes	deny	deny	deny	yes	yes	yes	deny	deny	yes	Type III	The left 8th rib	thoracic vertebra	lumbar vertebra									
Pubmed		1990	Filipino	F. BRETT SHANNON	Multifocal Cystic T uberculosis of Bone	man	4	deny	deny	deny	yes	yes	yes	deny	deny	deny	deny	deny	deny	Type II	Left shoulder shoulder blade	ILR										
Pubmed		2011	Morocco	F.Dlimi	Multifocal bone tuberculosis: A case report	woman	60	deny	deny	deny	deny	yes	yes	deny	deny	deny	deny	deny	deny	Type II	bilateral shin bone											
Pubmed		1996	Pakistan	F.L.DICKINSON	Multifocal skeletal tuberculosis:Bone scan appearances	woman	27	deny	deny	yes	deny	yes	yes	yes	deny	deny	yes	deny	yes	Type III	Left 4,. 8 ribs	cervical vertebra	left side greater trochanter	offside Sacroiliac joint								
Pubmed		1996	Pakistan	F.L.DICKINSON	Multifocal skeletal tuberculosis:Bone scan appearances	man	28	deny	deny	yes	deny	yes	yes	yes	yes	deny	deny	deny	yes	Type III	Right ankle joint	offside Sacroiliac joint	offside greater trochanter	thoracic vertebra	Right rib	Right sacrum Iliac joint	Right thigh Bone neck	right crus of diaphragm ankle bone				
Pubmed		1996	India	F.L.DICKINSON	Multifocal skeletal tuberculosis:Bone scan appearances	man	42	deny	deny	yes	deny	yes	yes	yes	yes	yes	deny	deny	deny	Type III	lumbar vertebra	thoracic vertebra	offside Sacroiliac joint									
Pubmed		1996	India	F.L.DICKINSON	Multifocal skeletal tuberculosis:Bone scan appearances	man	42	deny	deny	yes	deny	yes	yes	yes	yes	yes	deny	deny	yes	Type III	lumbar vertebra	thoracic vertebra	offside Sacroiliac joint	Left sacrum Iliac joint	rib							
Pubmed		1996	UK	F.L.DICKINSON	Multifocal skeletal tuberculosis:Bone scan appearances	man	59	deny	deny	yes	deny	yes	yes	yes	deny	yes	deny	deny	deny	Type III	lumbar	Right hip										

																					vertebra	joint										
Pubmed		1996	India	F.L.DICKINSON	Multifocal skeletal tuberculosis:Bone scan appearances	woman	60	deny	deny	yes	deny	yes	yes	yes	yes	yes	deny	deny	deny	Type III	lumbar vertebra	thoracic vertebra	Left sacrum Iliac joint									
Pubmed		2006	Turkey	Fazıl Gelal	Multifocal skeletal tuberculosis involving the lumbar spine and a sacroiliac joint: MR imaging findings	man	70	deny	deny	yes	deny	yes	yes	yes	deny	yes	deny	deny	deny	Type III	offside Sacroiliac joint	lumbar vertebra	sacrum	ilium								
Pubmed		1983	UK	G.0.HOPKINS	Multiple joint tuberculosis presenting as HLA-B27 disease	man	19	deny	deny	yes	deny	yes	yes	yes	deny	yes	deny	deny	deny	Type III	lumbar vertebra	Left knee joint										
Pubmed		2012	India	Gosal GS	Multifocal Skeletal Tuberculosis Involving the Lumbar Spine and Iliac Bone, Mimic king a Malignant Bone Tumour: A Case Report	man	25	deny	deny	yes	deny	yes	yes	yes	deny	yes	deny	yes	deny	Type III	lumbar vertebra	left side ilium	ischium									
Pubmed		2015	Iran	Hamidreza Haghighatkhah	Multifocal Skeletal Tuberculosis Mimicking Langerhans Cell Histiocytosis in a Child: a Case Report With aLong-Term Follow-Up	woman	5	deny	yes	yes	yes	yes	yes	yes	yes	yes	deny	deny	deny	Type III	two flanks frontal bone	Right shoulder shoulder blade	left ilium	left side pubis	thoracic vertebra	lumbar vertebra						
Pubmed		2017	USA	Hans A.Reyes	Multifocal Osteoart icular Tuberculosis in a Lupus With Sjogren Syndrome Patient	woman	41	unknown	deny	deny	deny	yes	yes	deny	deny	deny	deny	deny	deny	Type II	offside shin bone	Right hip joint	knee－joint	system Sexred Lupus								

Pubmed		2019	Indonesia	Heda Melinda Nataprawira	Multifocal Osteomyelitic Tuberculosis at Rare Locations with Metastatic Tu berculosis Abscess	woman	9	yes	deny	deny	yes	deny	yes	deny	deny	deny	deny	deny	deny	Type II	The fifth metacarpal bone of the left hand	Left ulna										
Pubmed		2003	Turkey	Hurrem Bodur	Multifocal tuberculosis presenting with osteoarticular and breast involvement	woman	40	deny	deny	deny	yes	yes	yes	deny	deny	deny	deny	deny	deny	Type II	Left ulna	Left radius	Left calcaneus	Left calcaneus	Left ankle joint	Tibial and plantar joint						
Pubmed		2021	Tunisia	Imen Chabchoub	Multifocal bone tuberculosis simulating metastasis	woman	22	deny	deny	yes	deny	yes	yes	yes	deny	yes	yes	yes	deny	Type III	cervical vertebra	lumbar vertebra	sacral	Left pubic bone	sternum							
Pubmed		2005	Turkey	Is¸lk Keles	Multifocal osteoarticular tuberculosis: a case report	woman	19	yes	deny	yes	deny	yes	yes	yes	yes	yes	yes	deny	deny	Type III	offside ilium	offside Sacroiliac joint	thoracic vertebra	lumbar vertebra	cervical vertebra							
Pubmed		2020	Colombia	Isabel Cristina Ramírez-Sánchez 1	Multifocal skeletal tuberculosis with mycobacteremia afterkidney transplantation: A case report	man	63	yes	yes	deny	deny	yes	yes	deny	deny	deny	deny	deny	deny	Type II	offside thigh-bone	frontal bone	orbital bone									
Pubmed		1988	India	K.Kumar	Multifocal osteoarticul ar tuberculosis	woman	4	deny	deny	deny	yes	yes	yes	deny	deny	deny	deny	deny	deny	Type II	Left knee joint	ossa extremitatis superioris	ossa extremitatis inferioris									
Pubmed		1988	India	K.Kumar	Multifocal osteoarticul ar tuberculosis	man	31	deny	deny	deny	yes	yes	yes	deny	deny	deny	deny	deny	deny	Type II	Left index finger	ankle	Right little finger	Right elbow joint								
Embase scopus wos		2019	Portugal	Lemos, Ana Pereira	Multifocal Chronic Osteomyelitis to Multiresistant Serratia Marcescens in a Child with Sick Cell Disease and Bone Tuberculosis	woman	12	deny	deny	yes	yes	deny	yes	yes	deny	yes	deny	deny	deny	Type III	humerus	radius	lumbar vertebra	sacral								
Pubmed		2010	China	Liang Hong	Multifocal skeletal tuberculosis: Experience in diagnosis and treatment	man	29	deny	deny	yes	yes	deny	yes	yes	yes	deny	yes	deny	yes	Type III	bilateral spealbone	thoracic vertebra	radix arcus vertebrae	Right 8、9、10 ribs	The left 12th rib	sternum	clavicalis	cervical vertebra	offside ischium	two ilium		
Pubmed		2016	China	LIANG ZHAN	Multifocal skeletal tuberculosis: A case report	man	19	deny	deny	yes	deny	yes	yes	yes	yes	yes	deny	yes	yes	Type III	lumbar vertebra	sacral	left ilium	thoracic vertebra	rib	Sacroiliac joint						
Pubmed		2013	Pakistan	Liaqat Ali Chaudhry	A 35-year-old immuno-competent male with open pulmonary tuberculosis associated with extra-ordinaryextensive extra-pulmonary tuberculosis	man	35	yes	deny	yes	deny	yes	yes	yes	yes	yes	deny	deny	deny	Type III	lumbar	thoracic	Left hip joint	bilateral Sacro	Right hip joint							

																					vertebra	vertebra		iliac joint								
Pubmed		2004	Pakistan	M.Ali	qy_Symmetrical lytic lesions in ulna unusual presentationof multifocal osteoart	woman	25	deny	deny	deny	yes	deny	yes	deny	deny	deny	deny	deny	deny	Type II	bilateral ulna											
Pubmed		2001	Turkey	M.TURGUT	Multifocal extensive spinal tuberculosis (Pott's disease) involving	woman	53	deny	deny	yes	deny	deny	deny	yes	yes	yes	yes	deny	deny	Type I	cervical vertebra	thoracic vertebra	lumbar vertebra									
Pubmed		2001	South Africa	Mahomed	ilhidl bb<br>Osseous Manifestations of Tuberculosis in Children	woman	1	unknown	deny	deny	yes	yes	yes	deny	deny	deny	deny	deny	deny	Type II	humerus	thigh-bone	shin bone									
Pubmed		2001	South Africa	Mahomed	Osseous Manifestations of Tuberculosis in Children	man	3	unknown	deny	deny	yes	deny	yes	deny	deny	deny	deny	deny	deny	Type II	humerus	radius										
Pubmed		2001	South Africa	Mahomed	Osseous Manifestations of Tuberculosis in Children	man	3	unknown	deny	deny	deny	yes	yes	deny	deny	deny	deny	deny	deny	Type II	first metatarsal bones	centrale										
Pubmed		2001	South Africa	Mahomed	Osseous Manifestations of Tuberculosis in Children	man	4	unknown	deny	deny	yes	deny	yes	deny	deny	deny	deny	deny	deny	Type II	big toe metacarpal bone	near-end Phalanges										
Embase scopus wos		2009	Iran	Majid Assadi	Diagnostic role of whole bo dy bone scintigraphy in atypical skeletal tuberculosis resembling multiple metastases: a case report	man	73	deny	yes	yes	yes	yes	yes	deny	deny	deny	deny	deny	yes	Type II	Right occipital parietal area	Right orbit frontal area	Left clavicle	Right thorax Subcondylar margin	Right thorax regiones claviculares	rib	Right humerus	Right femur	Right tibia	Right ankle joint	Right 1st One toe articulationes digitorum manus	left anterior arm, artifice, Hand

Pubmed		2004	Turkey	Mehmet Halit Yılmaz	Multifocal Skeletal Tuberculosis	man	24	deny	deny	yes	deny	deny	yes	yes	deny	deny	yes	deny	yes	Type III	cervical vertebra	left side fifth rib									
Pubmed		2004	Turkey	Mehmet Halit Yılmaz	Multifocal Skeletal Tuberculosis	woman	53	unknown	deny	yes	deny	yes	yes	yes	yes	yes	deny	yes	deny	Type III	lumbar vertebra	thoracic vertebra	sacral	offside Sacroiliac joint							
Embase scopus wos		2016	China	Meiping Ye	Multifocal musculoskeletal tuberculosismimicking multiple bone metastases: a case report	man	63	deny	deny	yes	yes	yes	yes	yes	yes	deny	yes	yes	yes	Type III	cervical vertebra	thoracic vertebra	bilateral clavicalis	bilateral rib	ilium	sacrum	pubis	articulatio coxae	thigh-bone		
Embase scopus wos		2019	Poland	Michał Znajdek	Juvenile spondyloarthritis and chronic recurrent multifocal osteomyelitis overlap syndrome in a16-year-old adolescent. A case report and literature review	woman	16	deny	deny	yes	yes	yes	yes	deny	deny	deny	deny	deny	deny	Type II	Left knee joint	left crus first Metatarsophalangeal joint	Right thorax Lock the joint	offside ilium	offside Iliac acetabulum	offside pubis	left side thigh-bone	offside perone	right crus of diaphragm first metatarsal bones		
Pubmed		2021	Iran	Milad Mellat-Ardakani	Multifocal Osteolytic Lesions in Skull Bone with MycobacteriumTuberculosis: A Case Report	man	37	deny	yes	deny	deny	deny	deny	deny	deny	deny	deny	deny	deny	Type II	bilateral maxillary alveolar ridge	bilateral maxillary protrusion	maxillary The base of the sinus	bilateral frontal sinuses	sphenoid bone						
Pubmed		2011	UK	Myo M. Lynn	Troublesome Tuberculosis: A Case Report onMulti-focal Tuberculous Osteomyelitis in An Immunocompetent Patient	man	52	deny	deny	yes	yes	yes	yes	deny	deny	deny	deny	deny	yes	Type II	rib	left side knee-joint	the right hand articulatio carpi	Left centre near-end fingerweb joint	Left ankle joint						
Pubmed		2020	Morocco	Naoufal Elghoul	A report of two challenging cases of bone infection: Mycobacterium tuberculosi s. How to manage?	man	81	deny	deny	deny	yes	deny	yes	deny	deny	deny	deny	deny	deny	Type II	bilateral humerus										
Pubmed		2017	India	Nitin M Rathod	Multifocal Tuberculosis Presen ting as Metastatic Tumour	woman	50	deny	deny	yes	deny	yes	yes	yes	yes	yes	deny	deny	yes	Type III	thoracic vertebra	fl	rib	lumbar vertebra							
Pubmed		2016	India	Pooja Dewan	Multifocal tuberculous osteomyelitis in a 3-yearold child	woman	3	deny	deny	deny	yes	deny	yes	deny	deny	deny	deny	deny	deny	Type II	bilateral humerus										
Pubmed		2015	India	Prasanna Chandiralingam	Multifocal tuberculous osteomyelitis: A rare presentation	woman	29	deny	deny	deny	deny	yes	yes	deny	deny	deny	deny	deny	deny	Type II	ILR	Right femur	offside Anterior iliac Upper spine								
Pubmed		2021	India	Rashmi Singh	Multifocal Osseous Tuberculosis Mimick ingLangerhans' Cell Histiocytosis: A Case Series	man	0.6	yes	yes	yes	deny	deny	deny	yes	yes	deny	deny	deny	deny	Type III	manubrium ster	thoracic verte	harnpan								

																					ni	bra										
Pubmed		2021	India	Rashmi Singh	Multifocal Osseous Tuberculosis Mimick ingLangerhans’ Cell Histiocytosis: A Case Series	man	3	yes	yes	deny	deny	yes	yes	deny	deny	deny	deny	deny	deny	Type II	left side frontal bone	parietal bone	Mastoid bone	left side ilium	offside thigh-bone	basis cranii						
Pubmed		2021	India	Rashmi Singh	Multifocal Osseous Tuberculosis Mimick ingLangerhans’ Cell Histiocytosis: A Case Series	woman	8	deny	yes	yes	deny	deny	deny	yes	deny	yes	yes	yes	deny	Type III	Right parietal	cervical vertebra	lumbar vertebra	sacral								
Embase scopus wos		2020	India	Rohan Sardana	Tuberculosis of the axial skeleton mimicking malignancy	woman	29	deny	deny	yes	deny	yes	yes	yes	yes	yes	yes	yes	deny	Type III	lumbar vertebra	thoracic vertebra	sacrum	sternum	cervical vertebra	two flanks sacrum	two flanks ilium					
Embase scopus wos		1994	Israel	RUTH HARDOFF	Multifocal Osteoarticular Tuberculosis Resembling Skeletal Metastatic Disease	woman	12	deny	yes	yes	yes	deny	yes	yes	yes	yes	deny	deny	deny	Type III	Left elbow	lumbar vertebra	thoracic vertebra	Left shoulder	upper left jaw	Left temporal lower jaw joint	sternum					
Pubmed		2008	Tunisia	S.Hassen- Zrour	Dactylite tuberculeuse multifocale: à propos d’un cas Multifocal tuberculous dactylitis: A case report	woman	42	deny	deny	deny	yes	deny	yes	deny	deny	deny	deny	deny	deny	Type II	the right hand third phalanx	the left hand fourth phalanx	the left hand third phalanx									
Pubmed		2020	Morocco	Salah Oueriagli Nabih	Contribution of radiobiphosph onates bone scan in multifocal tuberculous osteomyelitis	man	86	unknown	deny	yes	yes	deny	yes	yes	yes	deny	yes	deny	deny	Type III	bilateral clavicalis	bilateral humerus	cervical vertebra	thoracic vertebra								

Pubmed		2012	Korea	Seong Woo	Atypical Disseminated Skeletal Tuberculosis MimickingMetastasis on PET-CT and M RI	man	25	yes	yes	yes	deny	yes	yes	yes	yes	yes	yes	deny	yes	Type III	cervical vertebra	two flanks ilium	rib	thoracic vertebra	lumbar vertebra	harnpan						
Pubmed		2020	Malaysia	Shiau Li Lim	Multifocal Osteoarticular Tuberculosis in a Systemic Lupus Erythematosus (SLE) patient: a rarity or an underdiagnos ed condition?	woman	35	deny	deny	deny	yes	yes	yes	deny	deny	deny	deny	deny	deny	Type II	the left hand metacarpal bone	Right knee joint										
Pubmed		2015	China	SHUANG HU	Multifocal osteoarticular tuberculosis of t he extremities in an immunocompetent young man without pulmonary disease: A case report	man	19	yes	deny	deny	yes	yes	yes	deny	deny	deny	deny	deny	deny	Type II	two flanks shin bone	two flanks perone	two flanks radius	two flanks ulna	Right elbow joint	Right wrist joint	Right finger Interarticular joint	Right metatarsal Interarticular joint	Second from left phalanx			
Embase scopus wos		2011	India	Shyam S	MULTIFOCAL OSTEOARTICULAR TUBERCULOSIS PRESENTING AS OLIGOARTICULAR CHRONIC JUVENILE ARTHRITIS	man	18	deny	deny	yes	deny	yes	yes	yes	yes	deny	deny	deny	deny	Type III	right crus of diaphragm tarsal bone	Right boat Phalanges	thoracic vertebra	Right ankle joint								
Pubmed		2015	Iran	Taghi Baghdadi	Multifocal tuberculos is of long bones in an immunocompetent child	man	2	deny	deny	deny	deny	yes	yes	deny	deny	deny	deny	deny	deny	Type II	offside far-end shin bone	Right ankle joint										
Pubmed		1992	Spain	V SORIANO	Multifocal bone tuberculosis in one AIDS patient	woman	22	deny	yes	deny	deny	yes	yes	deny	deny	deny	deny	deny	deny	Type II	harnpan	left side shin bone										
Embase scopus wos		2019	USA	Victoria K.Shanmugam	Retinal vasculitis with Chronic Recurrent Multifocal Osteomyelitis: a case report and review of the literature	woman	37	deny	deny	yes	yes	deny	yes	deny	deny	deny	deny	deny	deny	Type II	offside clavicalis	sternum	Sterno-clavicle joint									
Pubmed		2022	China	Yen-Chun Chiu	Single posterior approach for circumferential debridement and anter ior reconstruction using fibular allograft in patients wit h skipped multifocal spinal tuberculosis	man	46	unknown	deny	yes	deny	deny	deny	yes	yes	yes	deny	deny	deny	Type I	thoracic vertebra	lumbar vertebra										
Pubmed		2022	China	Yen-Chun Chiu	Single posterior approach for circumferential debridement and anter ior reconstruction using fibular allograft in patients wit h skipped multifocal spinal tuberculosis	woman	48	unknown	deny	yes	deny	deny	deny	yes	deny	yes	deny	deny	deny	Type I	lumbar vertebra											
Pubmed		2022	China	Yen-Chun Chiu	Single posterior approach for circumferential debridement and anter ior reconstruction using fibular allograft in patients wit h skipped multifocal spinal tuberculosis	man	50	unknown	deny	yes	deny	deny	deny	yes	yes	yes	deny	deny	deny	Type I	thoracic vertebra	lumbar vertebra										
Pubmed		2022	China	Yen-Chun Chiu	Single posterior approach for circumferential debridement and anter ior reconstruction using fibular allograft in patients wit h skipped multifocal spinal tuberculosis	man	57	unknown	deny	yes	deny	deny	deny	yes	deny	yes	deny	deny	deny	Type I	lumbar vertebra											

Pubmed		2022	China	Yen- Chun Chiu	Single posterior approach for circumferential debridement and anter ior reconstruction using fibular allograft in patients wit h skipped multifocal spinal tuberculosis	man	58	unknown	deny	yes	deny	deny	deny	yes	yes	yes	deny	deny	deny	Type I	thoracic vertebra	lumbar vertebra										
Pubmed		2022	China	Yen- Chun Chiu	Single posterior approach for circumferential debridement and anter ior reconstruction using fibular allograft in patients wit h skipped multifocal spinal tuberculosis	man	60	unknown	deny	yes	deny	deny	deny	yes	yes	yes	deny	deny	deny	Type I	thoracic vertebra	lumbar vertebra										
Pubmed		2022	China	Yen- Chun Chiu	Single posterior approach for circumferential debridement and anter ior reconstruction using fibular allograft in patients wit h skipped multifocal spinal tuberculosis	woman	63	unknown	deny	yes	deny	deny	deny	yes	yes	yes	deny	deny	deny	Type I	thoracic vertebra	lumbar vertebra										
Pubmed		2022	China	Yen- Chun Chiu	Single posterior approach for circumferential debridement and anter ior reconstruction using fibular allograft in patients wit h skipped multifocal spinal tuberculosis	man	66	unknown	deny	yes	deny	deny	deny	yes	yes	yes	deny	deny	deny	Type I	thoracic vertebra	lumbar vertebra										

Pubmed		2022	China	Yen- Chun Chiu	Single posterior approach for circumferential debridement and anter ior reconstruction using fibular allograft in patients wit h skipped multifocal spinal tuberculosis	woman	73	unknown	deny	yes	deny	deny	deny	yes	yes	yes	deny	deny	deny	Type I	thoracic vertebra	lumbar vertebra										
Pubmed		2022	China	Yen- Chun Chiu	Single posterior approach for circumferential debridement and anter ior reconstruction using fibular allograft in patients wit h skipped multifocal spinal tuberculosis	man	73	unknown	deny	yes	deny	deny	deny	yes	yes	deny	deny	deny	deny	Type I	thoracic vertebra											
Pubmed		2022	China	Yen- Chun Chiu	Single posterior approach for circumferential debridement and anter ior reconstruction using fibular allograft in patients wit h skipped multifocal spinal tuberculosis	woman	75	unknown	deny	yes	deny	deny	deny	yes	deny	yes	deny	deny	deny	Type I	lumbar vertebra											
Pubmed		2022	China	Yen- Chun Chiu	Single posterior approach for circumferential debridement and anter ior reconstruction using fibular allograft in patients wit h skipped multifocal spinal tuberculosis	woman	79	unknown	deny	yes	deny	deny	deny	yes	yes	yes	deny	deny	deny	Type I	thoracic vertebra	lumbar vertebra										
Embase scopus wos		2013	Japan	Yoshifumi Kimizuka	A case of skeletal tuberculosis and psoas abscess: disease activity evaluated using 18 F-fluorodeoxyglucose positron emissiontomography-computed tomography	man	77	deny	deny	yes	yes	deny	yes	yes	yes	yes	deny	deny	yes	Type III	bilateral clavicalis	thoracic vertebra	sternum	rib	lumbar vertebra							
all places		2003	China	Chen Chao	One case of multiple iliac bone tuberculosis misdiagnosed as bone tumor	man	22	deny	deny	deny	deny	yes	yes	deny	deny	deny	deny	deny	deny	Type II	Right hip bone	ILR										
CNKI		2012	China	Chen Miaohong	One case of rare and frequent bone tuberculosis	man	49	unknown	deny	yes	deny	deny	deny	yes	yes	yes	deny	deny	yes	Type III	thoracic vertebra	lumbar vertebra	The right 9th rib									
Vip		2004	China	Chen Minyuan	One case of rare multiple iliac bone tuberculosis was reported	man	22	deny	deny	deny	deny	yes	yes	deny	deny	deny	deny	deny	deny	Type II	Right hip bone	ILR										
CNKI		2023	China	Chen Tao	Multiple bone tuberculosis, left skull base, middle ear and bilateral cervical lymph node tuberculosis 1 case	man	31	deny	yes	yes	deny	yes	yes	yes	yes	deny	deny	deny	yes	Type III	Right 4th posterior rib	left side basion	thoracic vertebra	bilateral sacrum	left side pubis							
Vip		2016	China	Chen Yonglin 1	Clinical analysis of six cases of misdiagnosis of atypical multiple bone and joint tuberculosis	man	17	deny	deny	yes	deny	deny	deny	yes	yes	deny	yes	deny	deny	Type I	cervical vertebra	thoracic vertebra										
Vip		2016	China	Chen Yonglin 2	Clinical analysis of six cases of misdiagnosis of atypical multiple bone and joint tuberculosis	man	34	deny	deny	yes	deny	deny	deny	yes	deny	yes	deny	deny	deny	Type I	lumbar vertebra											
Vip		2016	China	Chen Yonglin 3	Clinical analysis of six cases of misdiagnosis of atypical multiple bone and joint tuberculosis	woman	23	deny	deny	yes	deny	deny	deny	yes	yes	yes	yes	deny	deny	Type I	cervical vertebra	lumbar vertebra	thoracic vertebra									
Vip		2016	China	Chen Yonglin 4	Clinical analysis of six cases of misdiagnosis of atypical multiple bone and joint tuberculosis	man	42	deny	deny	yes	deny	deny	deny	yes	yes	yes	yes	deny	deny	Type I	cervical vertebra	lumbar vertebra	thoracic vertebra									
Vip		2016	China	Chen Yonglin	Clinical analysis of six cases of misdiagnosis of atypical multiple bone and joint tuberculosis	man	28	deny	deny	yes	deny	deny	deny	yes	deny	yes	deny	yes	deny	Type I	lumbar	sacral										

				5																	vertebra											
Vip		2016	China	Chen Yonglin 6	Clinical analysis of six cases of misdiagnosis of atypical multiple bone and joint tuberculosis	woman	62	deny	deny	yes	deny	deny	deny	yes	deny	yes	deny	deny	deny	Type Ⅰ	lumbar vertebra											
CNKI		2010	China	Dong Aisheng	One case of multiple bone tuberculosis	man	18	deny	deny	yes	deny	yes	yes	yes	deny	yes	yes	deny	deny	Type Ⅲ	cervical vertebra	lumbar vertebra	offside calcaneus									
all places	1957	1959	China	Dong Jinxing	A case report of multiple cystic bone tuberculosis	man	54	yes	deny	yes	deny	deny	deny	yes	yes	yes	deny	yes	yes	Type Ⅲ	thoracic vertebra	lumbar vertebra	The left 1st rib	Right side 1,. 2、5, 6 ribs	right sacrum							
all places	1987	1989	China	Jia Ming Suo	A case report of multiple cystic bone tuberculosis	woman	2	yes	yes	deny	yes	yes	yes	deny	deny	deny	deny	deny	deny	Type Ⅱ	Left radius	Right pubic bone	offside cheekbone	occipital bone								

CNKI	1987	1987	China	Jiang Shengda	One case of multiple cystic bone tuberculosis	man	7	yes	deny	yes	deny	yes	yes	yes	yes	deny	deny	deny	deny	Type III	thoracic vertebra	left side shin bone	left side huckle-bone	left side calcaneus	left side iliac bone	left side pubis	left side thigh-bone					
Vip		2019	China	Li Bangyin	One case of rare recurrence and multiple bone tuberculosis	man	46	yes	yes	yes	deny	yes	yes	yes	yes	yes	yes	yes	yes	Type III	cervical vertebra	lumbar vertebra	thoracic vertebra	occipital bone	rib	bilateral iliac bone	sacral	petrous bone				
Vip		2014	China	Min Li	One case of aplastic anemia with multiple bone and joint tuberculosis	woman	65	deny	deny	yes	yes	yes	yes	yes	yes	deny	yes	yes	deny	Type III	cervical vertebra	thoracic vertebra	Right hip bone	right sacrum	Right humerus							
CNKI		1999	China	Liang Hanjie	One case of multiple bone tuberculosis was reported	man	71	unknown	yes	yes	deny	yes	yes	yes	yes	deny	deny	deny	yes	Type III	offside cheek bone	thoracic vertebra	Left 9th rib	bilateral pubis	Right femur							
all places		1989	China	Liu Shangzhi	A case report of multiple cystic bone tuberculosis	man	2	deny	deny	deny	yes	yes	yes	deny	deny	deny	deny	deny	deny	Type II	bilateral humerus	bilateral ulna	bilateral radius	bilateral thigh-bone	bilateral shin bone	bilateral pubis						
all places	1952	1958	China	Ma Liansheng	A case report of multiple pseudocystic tuberculous osteomyelitis	man	2	yes	yes	deny	yes	yes	yes	deny	deny	deny	deny	deny	yes	Type II	bilateral claviculis	bilateral rib	occipital bone	bilateral spealbone	bilateral ossa extremitatis superioris	bilateral ossa extremitatis inferioris						
Vip		2015	China	Qu Manying	Analysis and review of literature on diagnosis and treatment of multiple bone and joint tuberculosis	man	22	deny	yes	yes	yes	deny	yes	yes	yes	yes	yes	deny	deny	Type III	thoracic vertebra	lumbar vertebra	left side cheek bone	cervical vertebra	synovium tuberculosis	Elbow joint Tuberculosis						
Vip	2013	2014	China	Shi Guangcan	One case of multiple bone tuberculosis	woman	65	deny	deny	yes	yes	deny	yes	yes	yes	yes	deny	yes	yes	Type III	manubrium sterni	thoracic vertebra	There are shoulders shoulder blade	Right side 4,. 5 ribs	Left 6,. 9, 11 ribs	lumbar vertebra	sacral					
CNKI		2010	China	Song music	Whole-body bone imaging diagnosis of multiple bone tuberculosis 1 case	woman	46	deny	deny	yes	deny	deny	deny	yes	yes	yes	yes	deny	yes	Type III	cervical vertebra	thoracic vertebra	lumbar vertebra	The left sixth rib								
CNKI		1998	China	Song Luxin	One case of multiple cystic bone tuberculosis	man	60	deny	deny	yes	yes	yes	yes	deny	deny	deny	deny	deny	yes	Type II	Right patella	Right ulna	Left pubic bone	left ilium	Left 5 , 6 ribs	Right 1st rib						
CNKI		1994	China	Tang San Yuan	A case of multiple bone tuberculosis in the same side limb is reported	woman	28	unknown	deny	deny	deny	yes	yes	deny	deny	deny	deny	deny	deny	Type II	Left tibia	Left patella	Left foot huckle-bone	left side centrale								
CNKI		2006	China	Wang Junpeng	One case of multiple bone tuberculosis was reported	man	34	unknown	deny	yes	yes	yes	yes	yes	yes	deny	deny	deny	deny	Type III	Left ulna	Left tibia	thoracic vertebra									

CNKI	1987	1987	China	Wei Guofu	A case of multiple tuberculosis of the skull combined with tuberculosis of the lateral condyle of the left humerus, tuberculosis of the middle phalanx of the thumb of the right hand and tuberculosis of the skin of the eyelids is reported	man	11	unknown	yes	deny	yes	yes	yes	deny	deny	deny	deny	deny	deny	Type II	left side cheek bone	Left humerus	Left ankle bone	the right hand big toe median segment phalanx								
Vip		2019	China	Xie Longzhou	Analysis of diagnosis and treatment process of one case of multiple bone joint tuberculosis complicated with skin tuberculosis	woman	68	deny	yes	yes	yes	deny	yes	deny	deny	deny	deny	deny	yes	Type III	frontal bone	nasal bone	bilateral cheek bone	basion	exognathion	Left ulna	Left humerus	offside claviculis	The first rib on the left	thoracic vertebra		
Vip		2011	China	Xu Hua	One case of bone and joint tuberculosis and skin tuberculosis in children and review of literature	man	10	yes	deny	yes	yes	yes	yes	yes	yes	yes	deny	deny	deny	Type III	offside ilium	Right ankle bone	Left foot calcaneus	the right hand nameless Point near The phalanges	thoracic vertebra	lumbar vertebra						
Vip		2004	China	Yang Ling	Misdiagnosis report of multiple tuberculous arthritis	woman	43	deny	deny	deny	yes	yes	yes	deny	deny	deny	deny	deny	deny	Type II	Both knees joint	Double ankles joint	both hands fingerweb joint	Elbows joint								

Vip		2007	China	Yang Zhiyun	One case of multiple bone and joint tuberculosis in the skull base, sphenoid and saddle area and the whole body	man	7	yes	yes	deny	yes	deny	yes	deny	deny	deny	deny	deny	deny	Type II	Left clavicle	offside ulna	Right minor Point near The phalanges	basion								
CNKI		2013	China	Yu Yipin 1	Three cases of bone tuberculosis combined with multiple bone infarction are reported	woman	54	deny	deny	deny	deny	yes	yes	deny	deny	deny	deny	deny	deny	Type II	bilateral thigh-bone	bilateral shin bone	system Sex red yabbi Sore for 6 years									
CNKI		2013	China	Yu Yipin 2	Three cases of bone tuberculosis combined with multiple bone infarction	man	57	yes	deny	deny	deny	yes	yes	deny	deny	deny	deny	deny	deny	Type II	offside shin bone	offside thigh-bone	He has been drinking for 30 years									
CNKI		2013	China	Yu Yipin 3	Three cases of bone tuberculosis combined with multiple bone infarction are reported	woman	25	yes	deny	deny	deny	yes	yes	deny	deny	deny	deny	deny	deny	Type II	bilateral thigh-bone	bilateral shin bone	nephroma synthesize Three years of service									
CNKI		1992	China	Yue Jiabin	One case of multiple cystic bone tuberculosis was reported	man	5	unknown	deny	yes	yes	yes	yes	yes	deny	deny	yes	deny	yes	Type III	offside pubis	Right humerus	Right shoulder shoulder blade	offside perone	Right femur	cervical vertebra	Right 7th, 8th rib	Left 8th posterior rib	Left 2nd anterior rib			
CNKI		2009	China	Zhang Lixia	One case of multiple bone tuberculosis with nodular erythema as the initial manifestation	man	41	yes	deny	deny	deny	yes	yes	deny	deny	deny	deny	deny	deny	Type II	Double patella	left side thigh-bone	Right tibia									
CNKI	1991	1993	China	Zhao Xiuzhen	Multiple bone and joint tuberculosis with bilateral pleural effusion	man	40	deny	deny	yes	yes	yes	yes	yes	yes	deny	deny	deny	yes	Type III	thoracic vertebra	Right 5th and 6th ribs	Shoulders shoulder blade	Double Ilium Bone	Double pubic bone							
